# EMG feedback improves grasping of compliant objects using a myoelectric prosthesis

**DOI:** 10.1186/s12984-023-01237-1

**Published:** 2023-09-13

**Authors:** Jack Tchimino, Jakob Lund Dideriksen, Strahinja Dosen

**Affiliations:** Neurorehabilitation Systems, Department of Health Science and Technology, Aalborg University, Aalborg, Denmark

**Keywords:** Closed-loop control, Prosthesis, EMG feedback, Force feedback, Compliant objects

## Abstract

**Background:**

Closing the control loop in myoelectric prostheses by providing artificial somatosensory feedback is recognized as an important goal. However, designing a feedback interface that is effective in realistic conditions is still a challenge. Namely, in some situations, feedback can be redundant, as the information it provides can be readily obtained through hearing or vision (e.g., grasping force estimated from the deformation of a compliant object). EMG feedback is a non-invasive method wherein the tactile stimulation conveys to the user the level of their own myoelectric signal, hence a measurement intrinsic to the interface, which cannot be accessed incidentally.

**Methods:**

The present study investigated the efficacy of EMG feedback in prosthesis force control when 10 able-bodied participants and a person with transradial amputation used a myoelectric prosthesis to grasp compliant objects of different stiffness values. The performance with feedback was compared to that achieved when the participants relied solely on incidental cues.

**Results:**

The main outcome measures were the task success rate and completion time. EMG feedback resulted in significantly higher success rates regardless of pin stiffness, indicating that the feedback enhanced the accuracy of force application despite the abundance of incidental cues. Contrary to expectations, there was no difference in the completion time between the two feedback conditions. Additionally, the data revealed that the participants could produce smoother control signals when they received EMG feedback as well as more consistent commands across trials, signifying better control of the system by the participants.

**Conclusions:**

The results presented in this study further support the efficacy of EMG feedback when closing the prosthesis control loop by demonstrating its benefits in particularly challenging conditions which maximized the utility of intrinsic feedback sources.

## Background

Loss of the upper limb has far-reaching consequences and profound effects on a person’s life. Those affected are faced with difficulties when executing everyday tasks [1], are reluctant to return to their workplace [2, 3], have reduced social participation [4], and potentially also suffer from phantom limb pain [5]. The use of a myoelectric prosthesis can offer functional restoration of lost hand functions. Nevertheless, despite technological developments in the field, a significant number of people with amputations still choose to abandon their myoelectric prostheses [6].

One of the drawbacks of contemporary prostheses is that normally they do not provide explicit somatosensory feedback to the user. Only two commercial systems incorporate a simple feedback interface [7, 8], while the clinical utility of feedback for the use of prosthetic hands in daily life is still to be established [9]. However, past research has indicated that closing the prosthesis control loop by supplementing it with artificial somatosensory feedback can be beneficial for the performance of such devices, possibly improving control as well as strengthening the users’ sense of embodiment [1, 10]. Nevertheless, developing feedback that is effective in realistic conditions of prosthesis use is still an open challenge [9].

To close the control loop, the prosthesis state can be measured using embedded sensors and then conveyed to the user through mechanical (vibration motors, linear actuators) or electrical stimulation (invasive or non-invasive) [11]. The grasping force is selected as the most common feedback variable in the literature [12–14], as an appropriate force is critical for safe and stable grasping.

One of the challenges when establishing the benefits of supplementary feedback in realistic conditions is that users can estimate the prosthesis state also from incidental cues, for instance, by observing the prosthesis motion, listening to the sound of its motors, feeling the interaction forces through the socket, or relying on the sense of their muscle contraction (muscle proprioception). It has been established for quite some time [15, 16] and demonstrated more recently that such incidental feedback can indeed be exploited in prosthesis control [17] and even outperform supplementary feedback in certain conditions [18, 19]. The power of incidental cues is also implicitly indicated by the fact that many studies investigating the benefits of supplementary feedback employ some kind of sensory deprivation (e.g., with a blindfold or noise-canceling headphones [20]) to restrict the participants’ access to such cues and, thereby, force them to utilize the artificial feedback.

Blocking incidental cues is, nevertheless, a useful experimental approach when developing and evaluating different interfaces to establish if the users can perceive and interpret the supplementary feedback. This condition, however, does not correspond to the realistic usage of a prosthesis during daily life, wherein users would receive both supplementary and incidental feedback. In this case, supplementary feedback may be redundant if the user can acquire relevant information by observing incidental cues [18]. Therefore, evaluating a feedback interface while the user is free to also exploit incidental information can provide further insight into the role of feedback in the prosthesis control loop.

The effect of incidental feedback is especially pronounced when a prosthesis user grasps a compliant object, as the object deformation can provide rich information about the applied force. Indeed, most studies [12, 13, 21–24] in the literature have used rigid or brittle objects to assess the impact of feedback. Some studies have used compliant objects [20, 25, 26] but the participants were blinded and the experiment aimed to assess if they can recognize object stiffness rather than control grasping force. A study [27] demonstrated that electrotactile feedback improved force control while grasping compliant objects despite the participants not being blinded. However, the participants did not use a real prosthesis, and the grasping was simulated through a prerecorded movie. Therefore, conventional approaches to providing supplementary feedback in prostheses can be ineffective when grasping compliant objects in the presence of vision, because both feedback sources provide the same information (grasping force).

These considerations motivated the development of another approach to close the control loop in prosthesis interfaces using noninvasive tactile stimulation. In this approach, instead of conveying the grasping force, the feedback transmits the user’s own myoelectric signal that drives the prosthesis (so-called “EMG feedback” [28]). The tactile stimulation, therefore, conveys an internal signal generated by the prosthesis controller, which means that the feedback transmits information that cannot be assessed using incidental sources. As shown in previous studies [28, 29], EMG feedback allows for the predictive adjustment of muscle contraction, while the prosthesis is closing, to reach the desired grasping force upon contact. Thus, we hypothesized that this approach could improve the performance of prosthesis force control even in the case where the participants are provided with an abundance of visual cues.

Hence, the present study assessed the performance when participants used a myoelectric prosthesis with and without EMG feedback to precisely manipulate compliant objects. The assumption was that the participants would perform similarly in terms of success rate in both conditions, as they can use the visual observation to successfully accomplish the task when EMG feedback is not provided. However, thanks to the predictive control enabled by EMG feedback, the time required to complete the task was expected to be shorter when EMG feedback was delivered. Lastly, the participants were expected to generate less variable myoelectric signals and more consistent commands when receiving EMG feedback.

## Materials and methods

### Participants

Ten healthy able-bodied participants (30 ± 4.08 years) without experience in myoelectric control and one participant with upper limb amputation were recruited for this experiment. The latter was 50 years of age, had suffered a traumatic transradial amputation 10 years prior, had been a user of a simple myoelectric prosthesis for 8 years, and was also familiar with the use of EMG feedback for prosthesis control.

### Experimental setup

The experimental setup comprised the following components: 1) a multifunctional myoelectric prosthetic hand (Michelangelo, Ottobock, DE), with a proprietary controller and two dry EMG electrodes with embedded amplifiers (13E200, Ottobock, DE), 2) four C2 vibrotactors and a control unit (Engineering Acoustics Inc., USA), 3) 3 clothespins of different stiffness values from a standardized clothespin test (Rolyan), 4) a laptop (Lenovo ThinkPad P52, Intel Core i7 @2.60 GHz, 32 GB RAM), running Windows 10 Professional and an 18” computer monitor. A specially made 3D-printed mount was used to attach the prosthesis to the forearm of the able-bodied participants. The participants’ wrists were immobilized with a thermoplastic splint (ORLIMAN) to enforce isometric muscle contractions. A custom-made socket was made for the amputee participant. The prosthesis and the tactor control unit were connected to the computer using a Bluetooth connection and a USB cable, respectively. The software that controlled the experimental setup was implemented in MATLAB Simulink 9.3, using the toolbox for closed-loop human-manual control [30].

**Fig. 1:**
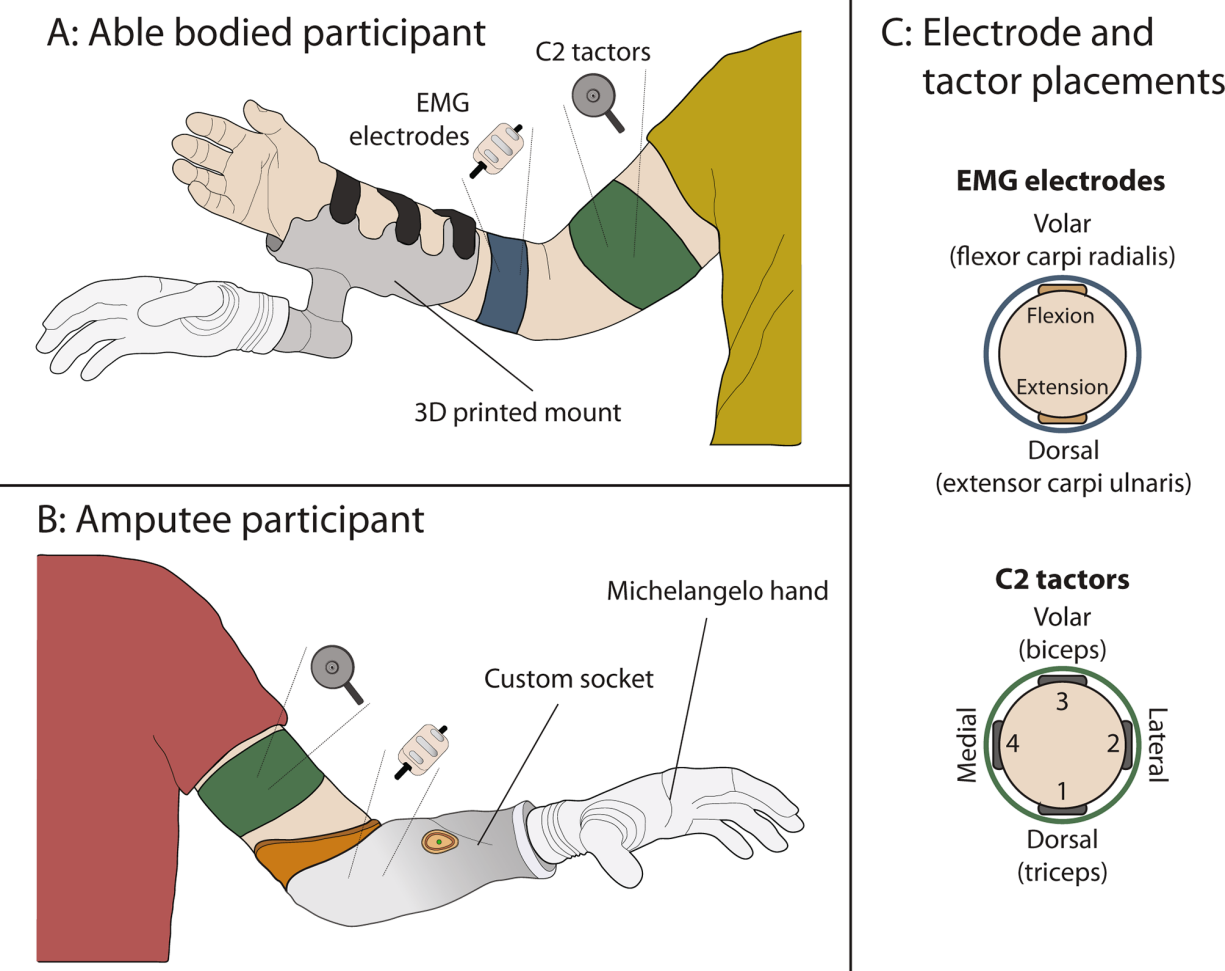
(**A**) An able-bodied participant wearing the EMG electrodes, C2 tactors, and the 3D printed mount with the Michelangelo hand attached. (**B**) The amputee participant wearing C2 tactors and the custom-made prosthetic socket with embedded EMG electrodes. (**C**) The placement of the EMG electrodes and the C2 tactors around the upper arm

The components and their placement on the participants are displayed in Fig. 1. The linear envelopes of EMG from the flexor carpi radialis and the extensor carpi ulnaris longus muscles (identified by palpation) were recorded by the EMG electrodes at 100 Hz and transmitted to the computer. The C2 tactors were placed circumferentially around the upper arm, approximately 5 cm proximal to the elbow.

The participants stood in front of a desk whose height was adjusted so that the participants could comfortably perform the experimental task. The clothespins setup was placed directly in front of them, while the monitor was positioned behind the clothespins, approximately 50 cm from the participants.

The Michelangelo prosthetic hand can perform two grasp types (palmar and lateral) and is also equipped with an active wrist module enabling hand rotation. Only hand opening and closing were controlled proportionally by a normalized command signal. If the prosthesis was open, the command signal set the desired closing velocity (motor speed). After contact with an object was established and the motor stalled, the command signal defined the grasping force. Therefore, the higher the command input, the faster the prosthesis closed (0 – no movement, 1 – maximum velocity) and the stronger it grasped the object (command input of 1 after contact – maximum force). Embedded sensors in the prosthesis measured the grasp force, which was transmitted to the laptop at a sampling rate of 100 Hz.

Importantly, as the present study used a compliant object, the prosthesis closing velocity corresponded to the transmitted velocity command only while the prosthesis moved freely. After the hand grasped the pins, the actual velocity was lower than that commanded by the participants, due to the opposing force that the pins applied on the fingers of the prosthesis. Nevertheless, after the hand stopped moving, either because it did not apply enough force to open a pin further or because the pin was fully open, the produced grasping force again corresponded to the prosthesis command input (myoelectric signal mapped according to Table 1).

**Table 1:** The processed EMG, desired closing velocity (prosthesis command), and force level thresholds. All signals are normalized, where 1 corresponds to the maximum closing velocity and grasping force

	**Lower thresholds**		
	**EMG**	**Velocity**	**Force**
Level 1	0.05	0	0
Level 2 – Target for **yellow** pin	0.2	0.15	0.2
Level 3 – Target for **green** pin, breakage for yellow pins	0.4	0.4	0.4
Level 4 – Target for **black** pin, breakage for yellow and green pins	0.65	0.75	0.75
Level 5 – Breakage for **all** pins	0.95	0.95	0.95

### Closed-loop myoelectric control

**Fig. 2:**
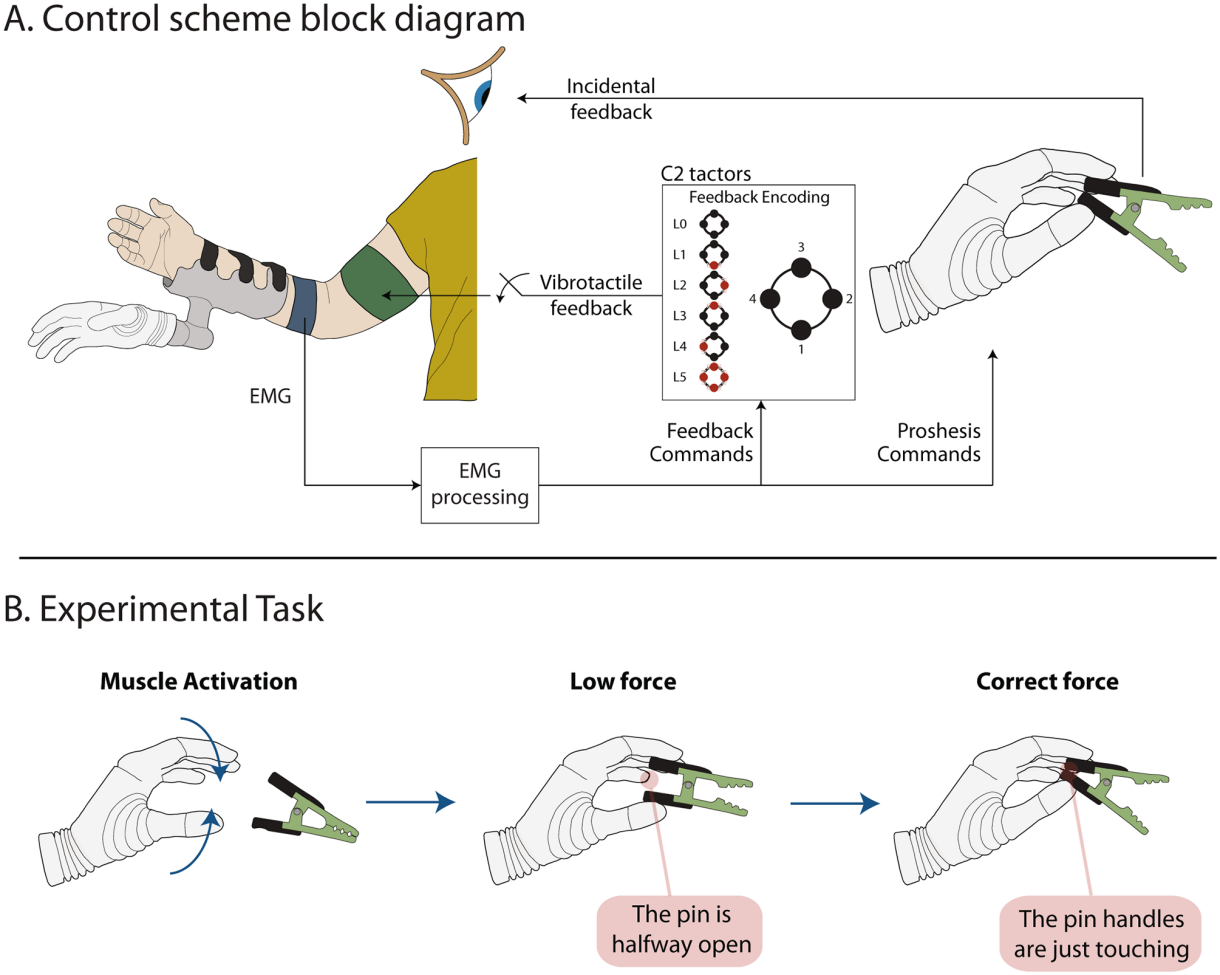
(**A**): The closed loop prosthesis control scheme. The linear envelope of EMG recorded by the electrodes was filtered and normalized, and then translated to prosthesis commands (continuous signal) and feedback levels (discretized). The tactors were activated to indicate the level of the myoelectric signal amplitude and each level was associated with a specific spatial stimulation pattern. The participants could use vision to monitor the movement of the prosthesis and closing of the pin. In the condition with no supplementary feedback, the EMG feedback was deactivated (switch). (**B**): The steps of the experimental task. The trial started with the participants generating a muscle activation which initiated the prosthesis movement. The task for the subjects was to grasp a pin and produce a force that was just enough to open it. Once the prosthesis grabbed a pin, a low force would open it only halfway. The correct force would open the pin fully, with the pin handles just touching. Further increase of the force would result in a failed trial

The design of the prosthesis control scheme is displayed in Fig. 2A and is similar to the one used in [21]. The myoelectric signal received from the prosthesis controller was normalized to 40% of the maximum voluntary contraction (MVC) and low pass filtered at 1 Hz using a 2^nd^-order Butterworth filter [31] (EMG processing block in Fig. 2). The myoelectric signal from the flexors was divided into six intervals (levels), using the thresholds: {0.05, 0.2, 0.4, 0.65, 0.95, 1}. These intervals were designed to be of increasing width, to mitigate the effect of the higher EMG variability in stronger muscle contractions [32]. The MVC normalization ensured that the prosthesis response and subjective experience when using the control interface were similar for all participants. In other words, the participants required the same percentage of their muscle strength to reach a specific myoelectric signal level and, thereby, close the prosthesis with the desired speed and generate the corresponding force, as explained below.

The normalized myoelectric signal (henceforth denoted simply as “EMG”) was then mapped to the prosthesis command input following a piecewise linear scheme, where the linear segments of the mapping were defined by the threshold values shown in Table 1. As explained in the previous section, the prosthesis command input controlled the prosthesis closing and grasping force and, therefore, the mapping in Table 1 established the proportional relation between EMG, closing velocity, and generated grasping force. The stronger the participant contracted the muscle, the faster the prosthesis closed and the higher the force generated when the prosthesis grasped a pin and stopped moving. The forces that were generated in response to the participants’ EMG are reported in the last column of Table 1. As expected, the normalized forces (column 3) corresponded to the normalized input commands (column 2). Since the hand opening was not relevant to the task, it was controlled by simply generating and maintaining an extensor contraction of over 0.6 on the normalized scale. The force measured by the embedded prosthesis sensors was also normalized (0 – hand is open, 1 – maximum grasp force).

Each participant performed the task with and without EMG feedback. The EMG feedback conveyed to the participant the momentary level of the myoelectric signal that they generated, as defined in Table 1. Each level was indicated by a specific activation pattern across the four vibrotactors. The first tactor was activated as soon as the myoelectric signal surpassed the dead zone. As the amplitude of the myoelectric signal increased from level 1 to 4, the individual tactors were activated sequentially in a counterclockwise manner. The highest level was represented by the simultaneous activation of all four tactors. This approach of conveying feedback information is known as spatial encoding and has been used extensively in literature because it is easy to perceive and interpret [22, 24, 29].

Note that although the feedback provided discrete information about the EMG level, the signal generated by the participant and transmitted as command input into the prosthesis was continuous. The participants could therefore still produce the closing velocities and grasping forces from the full working range of the prosthesis.

EMG feedback, therefore, enabled the participants to control the strength of their muscle contraction online. Since the myoelectric signal was proportional to the closing velocity and the grasping force, as explained above, the participants could use EMG feedback to control the prosthesis predictively and reliably. Specifically, the participants could adjust their contraction to the desired level during prosthesis closing, knowing that the force level they would apply after contacting the pin corresponds to the feedback they were receiving. For instance, if the EMG feedback indicated that the myoelectric signal was within level *n*, this would mean that the prosthesis would produce a gasping force of level *n* once fully closed. Levels n = 1…,5 are defined in Table 1 and, as explained before, the mapping was designed specifically to compensate for the higher EMG variability in stronger contractions, allowing the subjects to reach and maintain each level equally well.

**Fig. 3:**
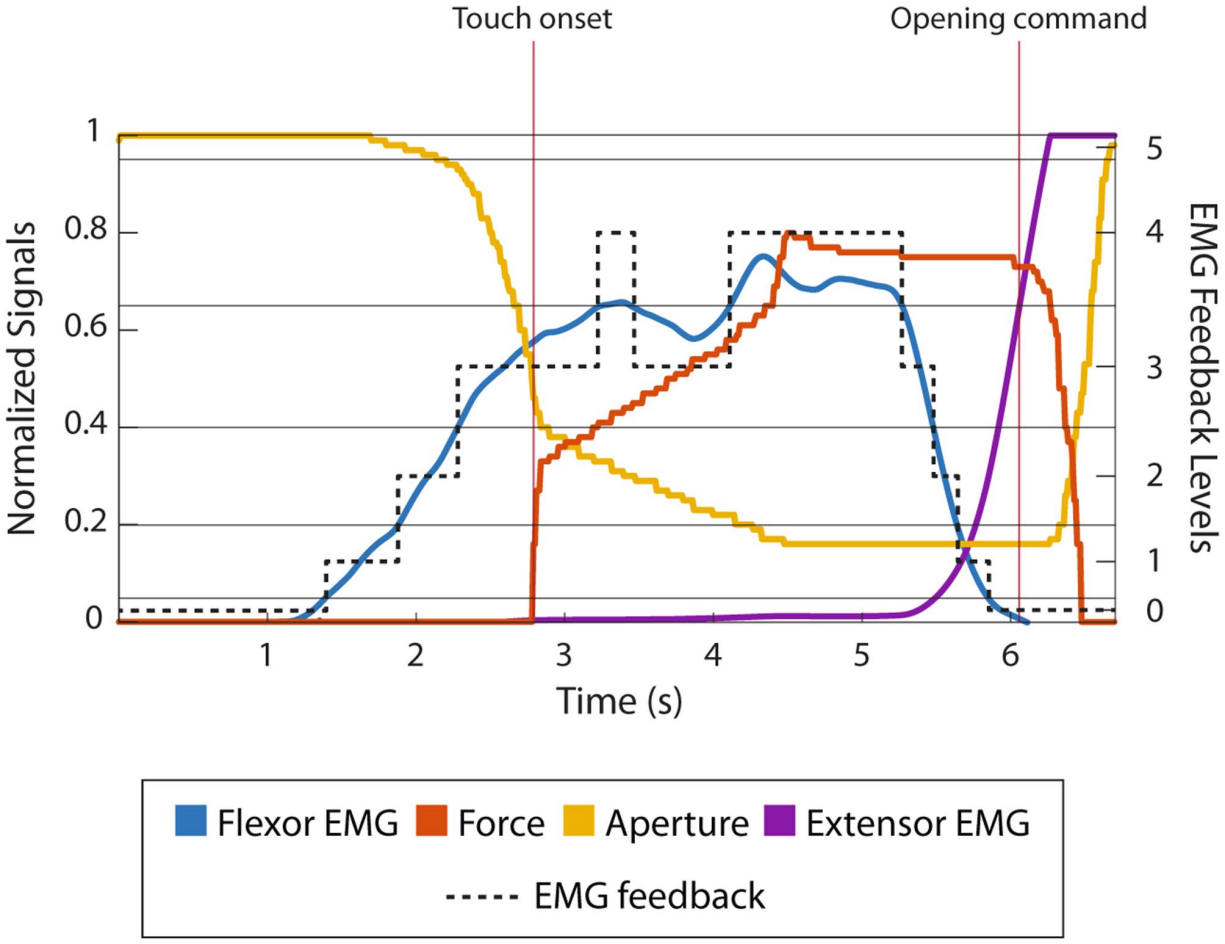
Representative trial to illustrate prosthesis control. The plot shows the traces for flexor (blue) and extensor EMG (purple), grasping force (red), prosthesis aperture (yellow), and EMG feedback level (dotted black line). The thresholds defining the levels are indicated as continuous thin black lines. The touch onset and opening command (trigger) are represented by the vertical red lines. The feedback signal (dotted black line) was conveyed to the participants online using vibration patterns defined in Fig. 2A.

A representative trial is shown in Fig. 3, to illustrate the prosthesis control interface. Initially, the prosthesis was fully open (aperture = 1), while the participant’s muscles were relaxed (EMG = 0). When they contracted their flexors, the corresponding EMG (shown in blue) was generated and mapped to the prosthesis control input (following the mapping in Table 1), which, then, activated the prosthesis, which started closing, thus decreasing the aperture (shown in yellow). The closing velocity (aperture slope) was proportional to the generated EMG. As soon as the prosthesis touched the pin (first vertical red line – “Touch Onset”), the hand started applying force (shown in red). Due to the opposing force from the pin, the prosthesis closing slowed down after the touch onset (less steep slope in the yellow line), despite the same amount of muscle activation. Like the closing velocity, the generated force was proportional to the EMG and the force gradually increased until it reached the EMG level the participant maintained. After the maximum force was applied (pin fully open), the participant relaxed their muscles and the flexor EMG dropped to zero; however, due to the non-backdrivability property of the prosthesis, it maintained the force exerted on the pin. When the participant activated their extensor muscles (second vertical line – “Opening Command”), the hand opened, letting go of the pin and the force then dropped to zero. The EMG feedback levels are represented by the dotted line and, as shown in the Figure, they indicated the range in which the flexor EMG lay at every moment, allowing the participant to anticipate the force that would be applied to the pin.

### Experimental task

Three clothespins of increasing stiffness values were selected through pilot testing, such that the forces required to open them fully did not overlap (see Table 1). The pins were colored yellow, green, and black, and were additionally fitted with 3D-printed components, which ensured consistent placement of the prosthesis fingers during grasping, thereby limiting the resulting moment arm, and improving the consistency of the task (i.e., ensuring reproducible force ranges to open each pin).

The task for the participants was to use the prosthesis to grab a pin and apply force to open it fully but without exerting a larger force than necessary to do so. Therefore, to succeed in the task, they needed to open the pin until the handles just touched (Fig. 2B). This would occur if the applied force corresponded to levels 2, 3, and 4, for the yellow, green, and black pins, respectively. The application of a larger force (e.g., level 3 when grasping a yellow pin) indicated that the compliant object was crushed and, thus, the trial was regarded as unsuccessful.

Therefore, the experimental task corresponded to the force-matching paradigm that is often used to assess the benefits of supplementary feedback [21]. However, this time, the participants were provided with an abundance of visual cues that they could use to perform the task successfully. For instance, they could see when the handles were about to touch and then carefully increase the force to produce a gentle contact, which would close the pin and produce the correct force. Finally, the participants could fail the task only by applying excessive force; hence, undershooting was not possible, as the pin had to be fully open for the trial to end.

### Experimental protocol

The setup was first calibrated for each of the participants. EMG signals generated when the participants were relaxed and when they generated an MVC were recorded. The former was used as the baseline signal that was subtracted from subsequent EMG recordings, while the latter was used for normalization, as explained before. The arm position during these recordings was the same as that which the participants assumed during the execution of the task.

The detection threshold (DT) was determined for each tactor by gradually increasing the intensity until the participants reported that they could feel the stimulation. The intensity used during the experiment was then set to DT + 0.4 × (MAX - DT), where DT is the detection threshold and MAX is the tactor’s maximum intensity. This value was selected since it elicited clear, localized, and non-intrusive sensations. Then, the vibrotactile patterns (Fig. 2A) associated with each feedback level were delivered to the participants in sequence, to demonstrate the sensations. A reinforced learning block followed, to ensure that the participants could recognize the feedback levels reliably. In this block, the vibrotactile patterns were presented in a randomized order and the participants were asked to identify the level associated with each pattern. The experimenter then disclosed the correct answer.

The main part of the experimental session was then conducted. The participants performed the task with and without EMG feedback, and the sequence of these two conditions was randomized across participants. In each condition, the participants first received two 30-trial training runs and then conducted four 30-trial experimental runs. The sequence of clothespins was pseudorandomized across participants. There were short 1-minute breaks between the runs in each feedback condition and a longer break between the conditions, to avoid muscle fatigue. During the training runs, the participants could see the target pin, trial number, and EMG and force traces on the computer monitor, with horizontal lines representing the threshold values associated with the feedback and force levels. The experimenter also explained the behavior of the system to the participants, pointing out how the traces on the monitor related to the movement of the prosthesis and the feedback (only in the EMG feedback condition) as well as to the task to be performed.

During the training in the condition without EMG feedback, the participants were instructed to observe the pin while grasping and try to open it just enough so that its handles touch, without applying more force than required to do so. Conversely, when EMG feedback was used, the correspondence between the EMG feedback and the applied grasping force was pointed out to the participants, who could then utilize the feedback to generate a precise force level that was sufficient to open the pin, again just enough (without squeezing it excessively). As explained in “*Experimental Task”*, the force level that effectively “solves” the task was determined by the pin color. In both cases, once the pin was fully open, the participants had to replace it on its rack by opening the prosthesis. The completion of each trial was denoted with a beeping sound from the laptop, which was delivered when detecting that the prosthesis had returned to its full aperture.

During the experimental runs, the EMG and force traces were not shown to the participants. The only information available to them was the force target in each trial and the trial number. Therefore, depending on the condition, they had to rely on incidental cues or a combination of incidental and EMG feedback to successfully complete the trials. Notably, the participants were not instructed to follow any particular strategy when performing the task.

### Data analysis

The main outcome measures were the success rate, defined as the percentage of trials in which the participants successfully accomplished the task and the trial completion time, as these two measures reflected the task performance. In addition, to characterize how the subjects generated the control input into the prosthesis, we computed the mean square jerk of the myoelectric signal within the trial, and the point-by-point variance (PPV) of the myoelectric signals across trials [33].

As explained previously, a trial was successful if the maximum attained force was within the target force level (see Table 1). The trial completion time was defined as the time from the moment t_1_ when the myoelectric signal crossed the dead zone (and the prosthesis started closing) to the moment t_2_ when the grasp force reached its highest value during the trial.

The same time range was used to compute the mean square jerk (MSJ) of the myoelectric signal. The MSJ indicates the signal’s “smoothness” [34] and was computed by:

1 $$MSJ = \frac{1}{{{t_2} - {t_1}}}\int_{{t_1}}^{{t_2}} {\mathop x\limits^{...} {{\left( t \right)}^2}dt}$$

where [t_1_ t_2_] is the trial time interval and $\stackrel{...}{x}\left(t\right)$ is the third derivative of the signal. A smoother signal corresponds to a lower MSJ value, and it can be viewed as an indication of better controllability when generating the commands.

Contrary to MSJ, which was computed for all trials, the point-by-point variance was computed considering only the successful trials. The trials were first normalized in time by resampling to 400 sample points. The variance of each sample over all trials was then calculated and the mean of the resulting values was used as the PPV. Lower PPV, therefore, indicated that a participant generated more consistent commands across trials.

The outcome metrics were computed for each participant for both feedback conditions and all three pin colors (stiffness values). The data from the amputee participant was not considered in the statistical analysis but is presented separately. The resulting datasets were tested for normality using the Lilliefors test. Depending on the outcome of the normality test, a two-way repeated measures ANOVA was applied to the datasets to calculate the main effects of the feedback type and stiffness, as well as their interaction. Non-normally distributed datasets were analyzed by comparing the feedback conditions for each stiffness, using Wilcoxon signed-rank tests. In addition, the results regarding the three stiffness values were compared within each feedback condition using Friedman tests and Wilcoxon tests for post-hoc comparisons. In both cases, the p-value was corrected using the Bonferroni method. StatSoft STATISTICA 8.0 was used for the statistical tests. The threshold for significance was set at p < 0.05 and the results are reported in the text as “mean ± standard deviation” (normal data) or “median {interquartile range}” (non-normal data).

## Results

**Fig. 4:**
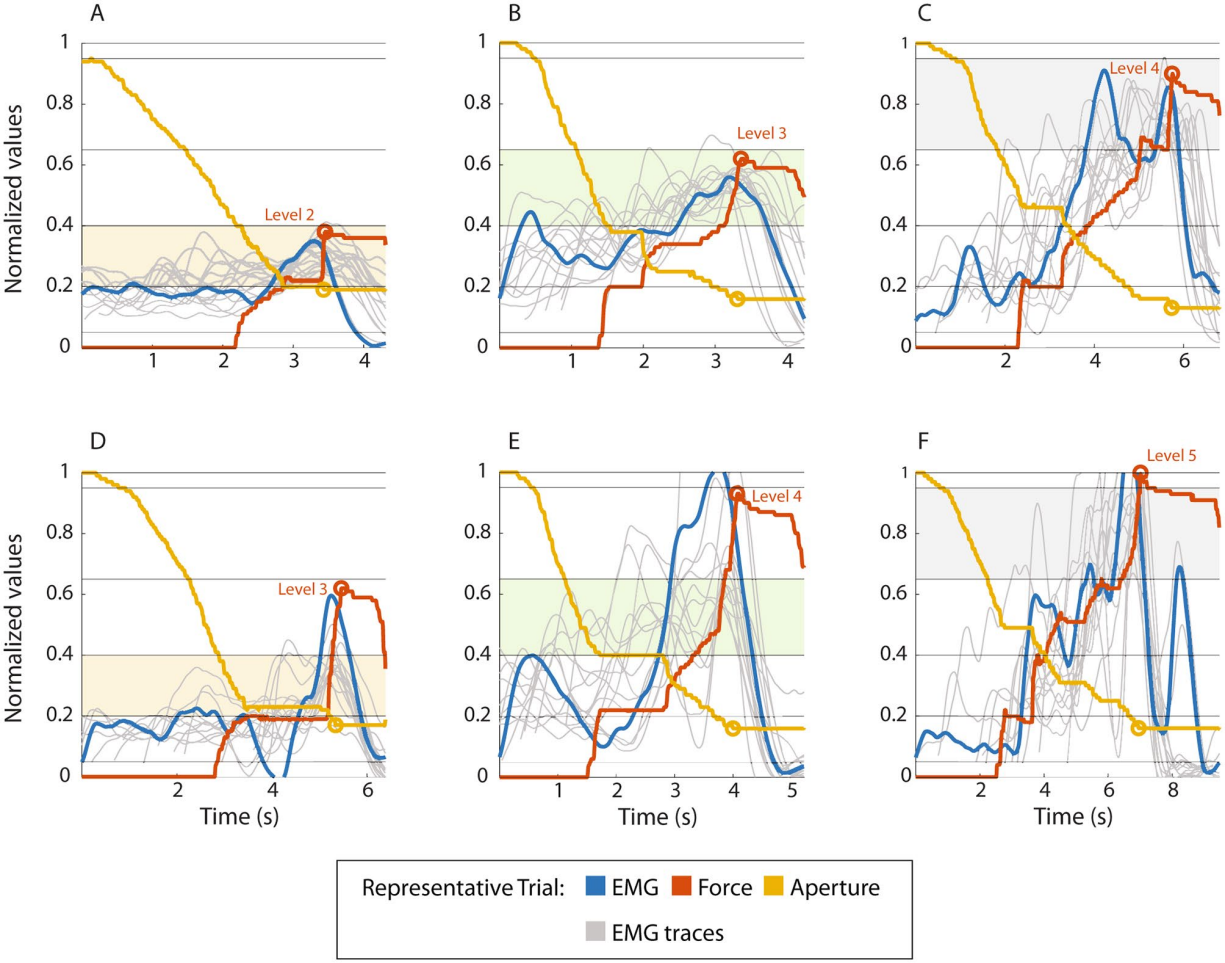
Representative experimental trials recorded in one participant (EMG, force, and prosthesis aperture). The EMG signals from other trials in the dataset are represented with the light grey lines. Plots **A-C** are the trials performed in the condition with EMG feedback, while plots **D-F** are trials conducted with incidental feedback only. The target force in each plot is highlighted by a color band and matches the color of the pin (yellow, green, or black). The maximum force in each plot is denoted by a red circle and the corresponding force level is also written next to the circle

Representative examples of the myoelectric signals generated when grasping pins with all three stiffness values with and without EMG feedback are shown in Fig. 4. As the participant activated their muscles (blue signal), the prosthesis started closing and the aperture decreased (yellow line). Once the fingers contacted the pin, the force signal appeared (red line). The closing then continued as the subject increased the muscle activation and thereby the prosthesis force. During this period, the subject could use visual feedback to assess how the pin handles approached each other until they finally touched (orange circle).

Notably, the force and EMG traces when EMG feedback was provided (Fig. 4A-C) display no overshoots, as opposed to those corresponding to the condition with incidental feedback only (Fig. 4D-F). An interesting observation is the adoption of a stepwise strategy for the completion of the task in both conditions, with the green and black pins (Fig. 4B, C, E, and F). This behavior was characterized by the initial application of a small force upon contact, a small pause (plateau in the yellow and red lines) followed by an upward modulation until the completion of the task.

The grey lines in Fig. 4 are the traces of the myoelectric signals generated by the same participant in other trials with the same target. Notably, by observing the morphology of the EMG signals across trials, it emerges that they are smoother and less variable when EMG feedback is available (Fig. 4A-C).

**Fig. 5:**
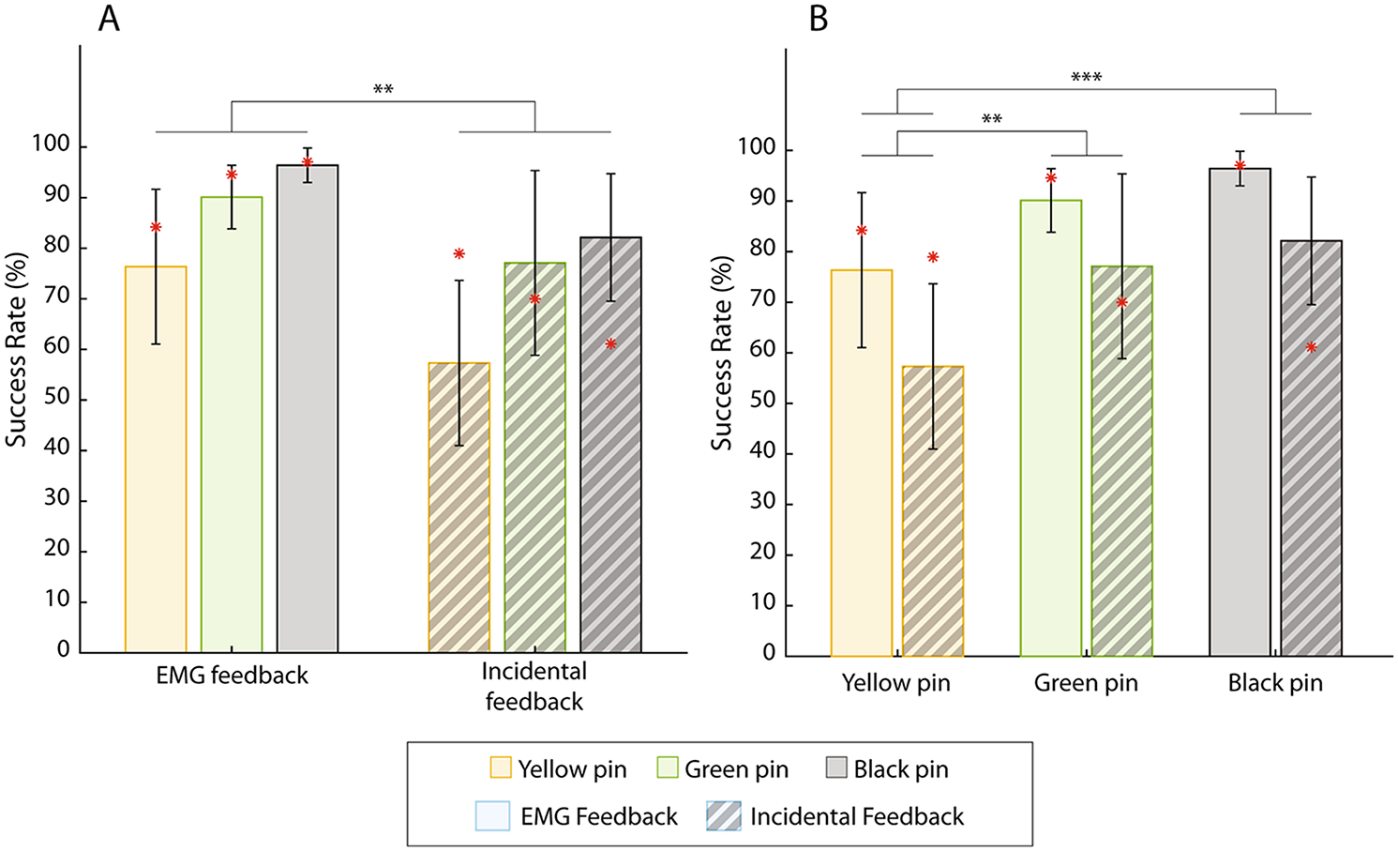
The success rates (mean ± standard deviation) grouped by feedback (**A**) and pin stiffness (**B**). The solid-colored bars correspond to the EMG feedback condition, while the striped bars correspond to the condition with incidental feedback only. The performance of the amputee participant is denoted by red asterisks. Statistically significant pairs are marked with horizontal bars (∗∗p < 0.01, ∗∗∗p < 0.001)

**Fig. 6:**
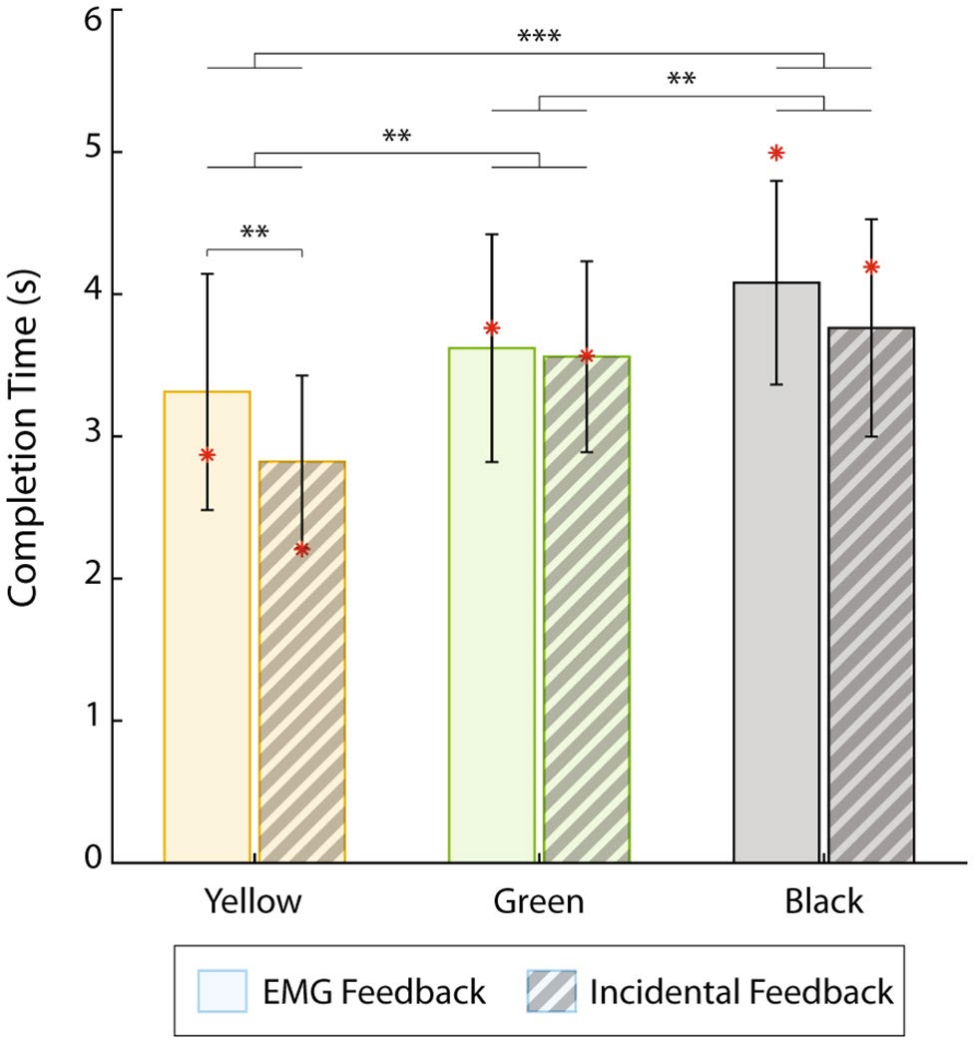
The completion time (mean ± standard deviation) for both feedback conditions, grouped by pin stiffness. The solid-colored and striped bars correspond to the conditions with EMG and incidental feedback, respectively. The performance of the amputee participant is denoted with red asterisks. Statistically significant pairs are marked with horizontal bars (∗∗p < 0.01, ∗∗∗p < 0.001)

**Fig. 7:**
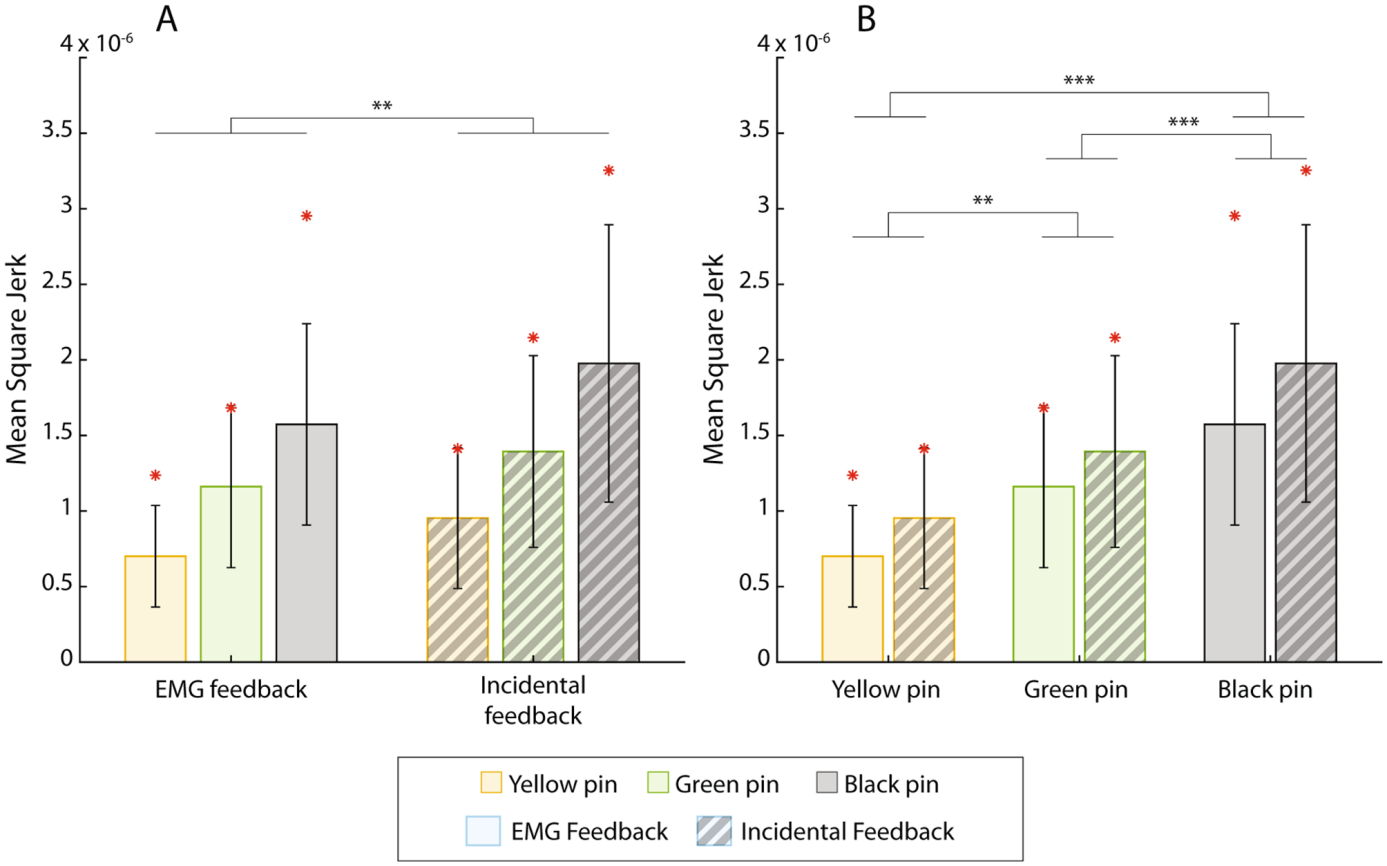
The mean square jerk (mean ± standard deviation) grouped by feedback condition (**A**) and pin stiffness (**B**). The solid-colored bars are for the EMG feedback condition, while the striped bars are for the condition with incidental feedback only. The performance of the amputee participant is denoted with red asterisks. Statistically significant pairs are marked with horizontal bars (∗∗p < 0.01, ∗∗∗p < 0.001)

**Fig. 8:**
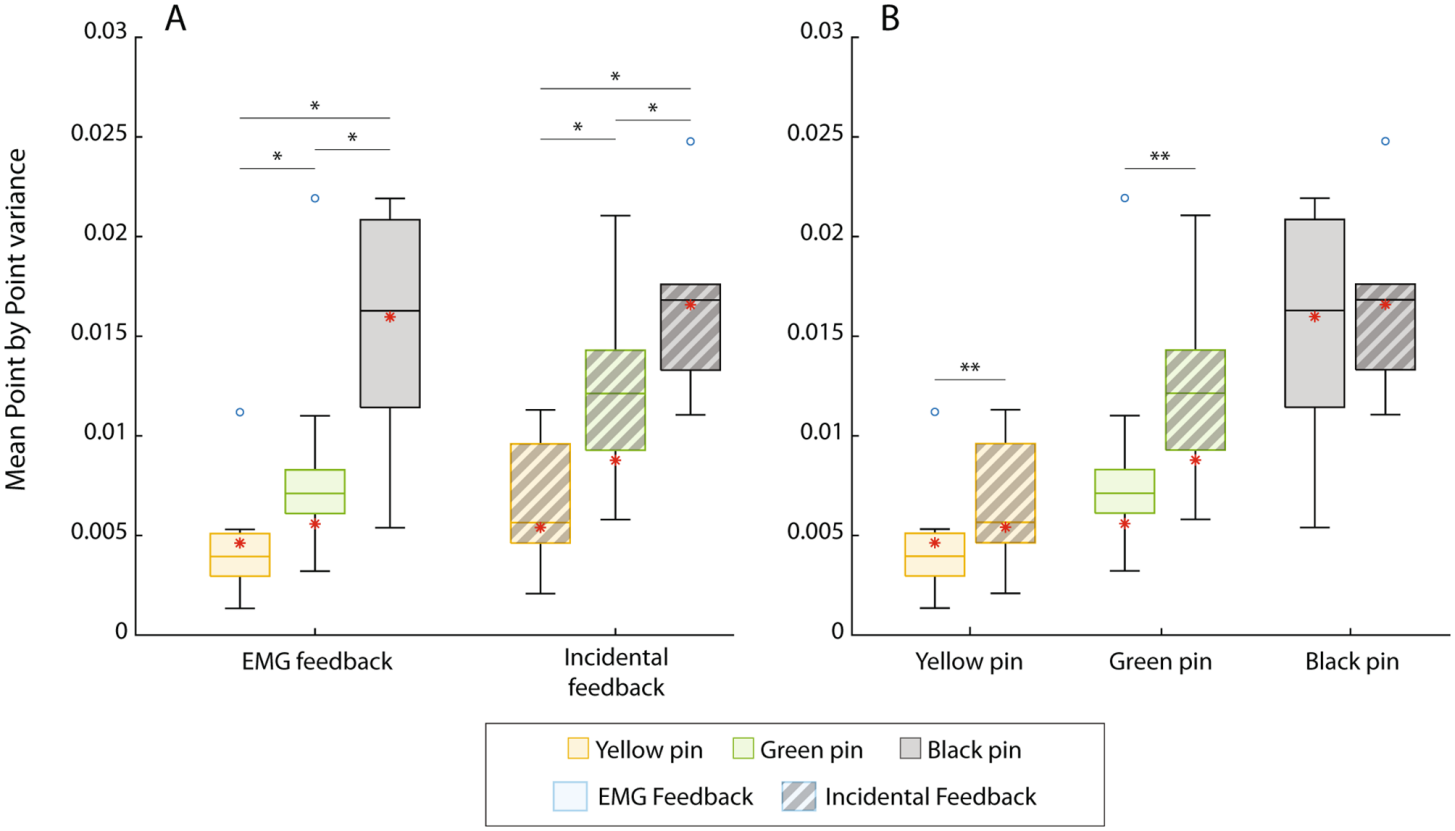
The boxplots for the point-by-point variance (PPV) grouped by feedback condition (**A**) and pin stiffness (**B**). The solid-colored boxes are for the condition with EMG feedback, while the striped boxes correspond to the condition with incidental feedback only. The performance of the amputee participants is denoted with red asterisks. Statistically significant pairs are marked with horizontal bars (∗p < 0.05, ∗∗∗p < 0.01)

The summary results for all subjects are shown in Figs. 5, 6, 7 and 8. The success rate is reported in Fig. 5. The statistical tests revealed that both the feedback (F[1, 9]= 16.418, p = 0.0029) and the stiffness (F[2, 18]= 16.521, p = 8e-5) were significant factors, with no significant interaction between them. The provision of EMG feedback significantly improved the performance over the condition with incidental feedback (87.6 ± 3.9% against 72.2 ± 5.8%), and the largest difference in the success rate was observed for the yellow pin (an increase of 19% with EMG feedback).

Regarding the trial completion time (Fig. 6), there was a significant (F[2, 18]= 4.51, p = 0.026) interaction between the factors. The post-hoc tests showed a slight but significant difference in the completion time between the two feedback conditions when grasping the yellow pin (3.31 ± 0.83 s for EMG feedback and 2.82 ± 0.6 s for incidental feedback). Therefore, surprisingly, the participants grasped the yellow pin slightly faster when only incidental feedback was available.

Both feedback and stiffness were significant factors regarding the MSJ (Fig. 7) ( F[1, 9]= 15.9, p = 0.003 for the feedback and F[2, 18]= 36.6, p = 4.4e-7 for the stiffness), while there was no significant interaction between them. The MSJ was consistently higher with incidental feedback only, while in both feedback conditions, higher stiffness values corresponded to higher MSJ. The post-hoc analysis showed that there were significant differences between all three stiffness pairs.

Due to a lack of normality, the PPV was compared between the two feedback conditions using Wilcoxon signed-rank tests for each stiffness value. The results regarding the PPV are shown in Fig. 8. The myoelectric signals were significantly less variable with EMG feedback when targeting the yellow (p = 0.028) and green (p = 0.02) pins compared to the condition with incidental feedback only. For both feedback conditions, the Friedman tests indicated that there was a significant difference in the PPV between stiffness values (χ^2^ [2]= 20 and p = 5e-5). The subsequent Wilcoxon tests showed that the PPV was significantly different (p = 0.015) between all pairs of stiffness values.

The results of the amputee participant are indicated in all figures using a red asterisk. Importantly, the performance between the conditions with and without EMG feedback follows the trends demonstrated by able-bodied subjects in all cases, except for the success rate across pins in the condition with incidental feedback (Fig. 5A). This is an encouraging result for the prospective clinical translation of the approach.

## Discussion

This study explored the benefits of EMG feedback when controlling the myoelectric prosthesis grasping force while handling a compliant object and compared it to the performance achieved when using only incidental feedback. The results indicated the advantage of EMG feedback over the incidental sources alone, even though the behavior observed did not fully correspond to our initial expectations.

Despite the abundance of visual cues provided by the deformation of the pins, the results demonstrated that EMG feedback increased the success rate in accomplishing the task. We assume that the supplementary feedback was beneficial because it provided information (myoelectric signal) that is useful for grasping force control, but which cannot be assessed using incidental feedback, as it is an internal signal computed by the prosthesis controller. Therefore, the feedback still enhanced and added to the information already provided by the incidental cues (the deformation of the pin indirectly conveyed the generated grasping force). This is in agreement with the observation in [18], where it is stated that the feedback should indeed deliver novel information if it should benefit the control over simple incidental cues. As shown in a recent study [19], vision and audition can provide very good estimates of the prosthesis state and if the tactile stimulation simply duplicates those variables, the impact is likely to be limited.

To perform the task without EMG feedback, the participants had to monitor the movement of the handles while opening the pins. In addition, during the final phase of the task, before the pin handles touched, they had to exercise fine control of their muscle activation in order not to squeeze the pin excessively. However, the myoelectric signal is variable and the prosthesis response to the increase in the signal can be abrupt. For instance, Fig. 4F shows a stepwise response of the force signal, despite a smooth modulation of the myoelectric input. Force control was, therefore, challenging, despite the visual cues; the latter did indicate the change in force but only after such a change had already happened. In summary, the lack of information about the command input generated by the participants, combined with occasionally abrupt prosthesis responses, caused them to perform inconsistent contractions and fail the task. This was confirmed by the secondary outcome measures, which showed that without EMG feedback the subjects generated less smooth signals within trials (lower MSJ, Fig. 7), as well as less consistent contractions across trials (lower PPV, Fig. 8), confirming the findings in [33].

The present study did not compare different feedback interfaces, for instance, EMG versus force feedback. Nevertheless, the information provided by force feedback can be estimated directly from the visual observation of the pins, as the applied grasping force corresponds to pin deformation. As stated in [18], artificial feedback is only useful if it provides information additional to the one already available to the user. Indeed, we believe that the effectiveness of EMG feedback is that it provided both force information (indirectly) and information about intrinsic control signals (directly), where the latter cannot be estimated through incidental cues.

When EMG feedback was provided, the participants could execute the task by activating their muscles to match the EMG feedback level they received online to the force level that corresponded to the target pin, thereby reducing their reliance on visual cues for the completion of the task. Ideally, if a participant generated and maintained a muscle contraction at a level that corresponded to the target force (pin color), the prosthesis would grasp the clothespin with an appropriate force and open it to its full aperture in one fluid motion. However, as shown in Fig. 4, the participants most often selected a different strategy. In both conditions, with and without EMG feedback, they approached the pin slowly by generating a myoelectric signal below the lower threshold of the target level. In such cases, the force applied by the hand would not suffice to fully open the pin and the fingers would stop moving (Fig. 4, red and yellow line plateaus) immediately after grasping the clothespin, or after the latter had been partly opened. In either case, to resume the task, the participants had to modulate their muscle contraction upwards, until the force applied by the hand was higher than the opposing force of the pin.

Without EMG feedback, this was not an easy task, as explained before. The hand was immobile, hence not producing incidental cues, while the only information about the control input into the prosthesis was an indirect and unreliable estimate of the myoelectric signal provided by the natural muscle proprioception. Therefore, the increase in muscle contraction was, in this case, performed “blindly” by the participants, and the response of the prosthesis was hard to predict accurately. Conversely, with EMG feedback, the participants always received information about the amplitude of their myoelectric signal, even when the prosthesis was not moving. This allowed them to better control the prosthesis and the pin opening and, hence, achieve a higher success rate in accomplishing the task.

Our initial expectation was that the participants would perform the task significantly faster when EMG feedback was provided. We assumed that they would adopt the strategy outlined above, wherein they would rely on EMG feedback to directly produce the force level associated with the pin color. However, the fact that EMG feedback did not lead to faster grasping, implies that the participants did not completely give up on the incidental cues. The final strategy was likely to assess and utilize both sources of information – the information on the control input (EMG feedback) and the prosthesis response (visual feedback). This was probably a conservative choice, where the participants opted for a safer approach, as a faster completion time might come with a tradeoff [33]. It remains to be investigated if the adopted strategy can be changed with training.

Importantly, the dominance of EMG feedback with respect to incidental sources was expressed across a range of stiffness values. The success rate was significantly higher consistently for all three pins, but the gain was largest for the softest pin. This is in accordance with the discussion so far, as in this case, the loss of control during force increase is more heavily penalized (easy to squeeze the pin and overshoot the force). The stiffer pins could still effectively “buffer” an uncontrollable increase in prosthesis force.

The number of levels of discrete feedback was based on prior literature [21, 31] and pilot tests. The advantage of discretized feedback is that it is easy to learn, while a drawback is its limited feedback resolution. Importantly, the present study shows that EMG feedback, even when discretized, leads to better performance despite the presence of rich incidental cues. Importantly, since the control was continuous, the participants could, in principle, use the feedback to achieve sub-level accuracy (e.g., reach a level and increase the contraction a little further to apply a higher grasping force, still within the same level); however, this was not tested in the present experiment. The implementation of a larger number of levels and the impact of this parameter on the control would be, of course, interesting to investigate in future studies.

The focus of the present study was to compare the feedback conditions while the difference in performance between the pins was a secondary observation. The consistent increase in the completion time, PPV, and MSJ from low to high stiffness was characteristic for both conditions (with and without EMG feedback) and can be explained by the higher forces required to open stiffer pins.

## Conclusions

Overall, the results of this study indicated that EMG feedback for the control of a myoelectric prosthesis can enhance the information estimated from incidental cues, even in the presence of an abundance of such cues. The provision of EMG feedback significantly improved the control performance when grasping compliant objects. Objects of three different stiffness levels were used, and the feedback was shown to benefit force control in all three cases. These outcomes constitute further proof of the effectiveness of EMG feedback and will, hopefully, pave the way for its eventual integration into prosthetic hands.

## Data Availability

The datasets used and/or analysed during the current study are available from the corresponding author upon reasonable request.

## List of abbreviations

DT: Detection threshold
EMG: Electromyography
MSJ: Mean square jerk
MVC: Maximum voluntary contraction
PPV: Point by point variance
SR: Success rate
